# Supplementary material for: Simultaneous isolation of proximal and distal lung progenitor cells from individual mice using a 3D printed guide reduces proximal cell contamination of distal lung epithelial cell isolations
Source: Stem Cell Reports. 2022 Dec 1;17(12):2718–31. doi: 10.1016/j.stemcr.2022.11.002 (PMC9768627; doi:10.1016/j.stemcr.2022.11.002)
Supplement: Document S2. Article plus supplemental information [file mmc4.pdf]

Stem Cell Reports
Resource
OPEN ACCESS

Simultaneous isolation of proximal and distal lung progenitor cells from individual mice using a 3D printed guide reduces proximal cell contamination of distal lung epithelial cell isolations

Hani N. Alsafadi,[1,2,3] John Stegmayr,[1,2,3] Victoria Ptasinski,[1,2,3,5] Iran Silva,[1,2,3] Margareta Mittendorfer,[1,2,3] Lynne A. Murray,[5,6] and Darcy E. Wagner[1,2,3,4,*]
[1]Department of Experimental Medical Sciences, Faculty of Medicine, Lund University, Lund, Sweden
[2]Wallenberg Centre for Molecular Medicine, Faculty of Medicine, Lund University, Lund, Sweden
[3]Stem Cell Center, Faculty of Medicine, Lund University, Lund, Sweden
[4]NanoLund, Lund University, Lund, Sweden
[5]Research and Early Development, Respiratory & Immunology, BioPharmaceuticals R&D, AstraZeneca, Gothenburg, Sweden
[6]Research and Early Development, Respiratory & Immunology, BioPharmaceuticals R&D, AstraZeneca, Cambridge, UK
*Correspondence: darcy.wagner@med.lu.se
https://doi.org/10.1016/j.stemcr.2022.11.002

SUMMARY

The respiratory epithelium consists of multiple, functionally distinct cell types and is maintained by regionally specific progenitor populations that repair the epithelium following injury. Several *in vitro* methods exist for studying lung epithelial repair using primary murine lung cells, but isolation methods are hampered by a lack of surface markers distinguishing epithelial progenitors along the respiratory epithelium. Here, we developed a 3D printed lobe divider (3DLD) to aid in simultaneous isolation of proximal versus distal lung epithelial progenitors from individual mice that give rise to differentiated epithelia in multiple *in vitro* assays. In contrast to 3DLD-isolated distal progenitor cells, commonly used manual tracheal ligation methods followed by lobe removal resulted in co-isolation of rare proximal cells with distal cells, which altered the transcriptional landscape and size distribution of distal organoids. The 3DLD aids in reproducible isolation of distal versus proximal progenitor populations and minimizes the potential for contaminating populations to confound *in vitro* assays.

INTRODUCTION

The respiratory epithelium consists of several distinct cell types distributed within the nasal and tracheal passages leading to the branching airways and terminating in the distal alveoli, where gas exchange occurs (Rock et al., 2010; Volckaert and De Langhe, 2014). Although the lung is known to have a limited regenerative capacity, it contains several region-specific progenitors that proliferate and subsequently differentiate to repair damaged lung epithelium (Chen et al., 2012). The cells responsible for epithelial repair have been described in several seminal papers depicting their location in the lung and their regenerative capacity following injury (Barkauskas et al., 2013; Kim et al., 2005; McQualter et al., 2010; Rawlins et al., 2009; Reynolds et al., 2000; Rock et al., 2009).

The tracheal epithelium is maintained by a heterogeneous pool of p63$^+$Krt5$^+$ basal cells (Rock et al., 2009), while the normal bronchioles and alveolar epithelium are maintained by Scgba1$^+$ cells (termed club cells) and Sftpc$^+$ alveolar type II (ATII) cells, the progenitor cell of the alveolus (Barkauskas et al., 2013; Rawlins et al., 2009). Additionally, subpopulations such as Itga6$^+$Itgb4$^+$ alveolar progenitors and bipotent progenitor populations such as bronchioalveolar stem cells (BASCs) (Scgb1a$^+$Sftpc$^+$) and Hopx$^+$ also give rise to both small airway and alveolar cell types, respectively (Chapman et al., 2011; Jain et al., 2015; Kim et al., 2005; Rawlins et al., 2009). Single-cell RNA sequencing (scRNA-seq) studies are continuously improving our understanding of the diverse cellular landscape in the lung, including progenitor and transitional populations that arise and expand during development and during normal aging and disease (Sikkema et al., 2022). These transitional states are defined by the co-expression of multiple phenotypic markers, which were previously thought to be mutually exclusive in defining differentiated or distinct progenitor populations. Therefore, hereafter, for simplicity, we will refer to the basal epithelial cells located in murine tracheas as proximal progenitors and those from parenchymal cell isolations as distal progenitors.

In vitro and *in vivo* approaches have been used to identify the cell types involved in lung repair and regeneration. *In vivo* lineage tracing has been instrumental in identifying several region-specific progenitor cells (Tata and Rajagopal, 2017), but the promotor regions used to mark these populations are known to be leaky, and scRNA-seq studies have further confirmed that multiple and distinct cell types express these markers, even in healthy lungs and airways (Joshi et al., 2020; Montoro et al., 2018; Perl et al., 2009; Rawlins, 2008; Strunz et al., 2020). Thus, an increasing number of studies have used organoids derived from primary lung epithelial progenitor cells to identify and monitor the cell populations responsible

This is an open access article under the CC BY license (http://creativecommons.org/licenses/by/4.0/).

for repair and regeneration in the lung epithelium (Barkauskas et al., 2017). However, the proportion of proliferating cells in this assay is known to be small and is highly dependent on the ability to isolate well-defined progenitor populations.

Several techniques exist to isolate lung epithelial progenitors from the trachea/proximal airways or parenchymal tissue (Dobbs et al., 1986; Jansing et al., 2018; Lam et al., 2011; Vanderbilt et al., 2015; You et al., 2002), but a protocol to simultaneously and specifically isolate distal (e.g., ATII cells) and proximal progenitors (basal cells) from individual murine lungs has not been described, because of a lack of unique surface marker(s) distinguishing these cells (Kim et al., 2005; Montoro et al., 2018; Rawlins et al., 2009). Isolation of tracheal epithelial cells is done by surgically resecting the trachea and incubating with dissociation reagents followed by media selection or flow or magnetic associated cell sorting to enrich for basal cells. On the other hand, most approaches for distal epithelial cell isolations use instillation of dissociating agents through the trachea, followed by ligation of the trachea or, less commonly because of technical challenges, at the level of the main bronchi (Jansing et al., 2018; Messier et al., 2012). Single-cell suspensions are then collected from the parenchymal tissue to isolate the distal epithelial progenitors, often through epithelial cell adhesion molecule (EpCAM) positivity or through fluorescently labeled reporter mice for specific phenotypic markers (e.g., *Sftpc* promoter). However, the success of these approaches is variable, with some reports finding only 25%–60% of ATII cells captured using *Sftpc*-based labeling (Chen et al., 2012; Perl et al., 2009; Vanderbilt et al., 2015). Furthermore, *Sftpc* is expressed in multiple cell types of the airway epithelium and confirmed in recent scRNA-seq datasets (Perl et al., 2009; Rawlins, 2008; Strunz et al., 2020), calling into question its specificity for ATII cell isolations. On the other hand, $EpCAM^+$ selection is commonly used, occasionally in combination with additional surface markers (e.g., Sca1), to demarcate different regenerative subpopulations (Louie et al., 2022; McQualter et al., 2010). However, both proximal and distal epithelial progenitors express EpCAM, and thus a small number of proximal cells may be inadvertently co-isolated in such approaches. This is especially problematic in organoid studies where around 0.5–2% of cells are identified as giving rise to organoids (Barkauskas et al., 2017).

Understanding the response and expansion of regionally specific progenitor cells in normal lung development, homeostasis and disease is an area of active and ongoing investigation (Sikkema et al., 2022; Vaughan et al., 2015; Zuo et al., 2015). Increases in proximal-associated phenotypic markers has been observed in the distal portions of diseased lung tissue, but the origin of these cells remains incompletely understood (Vaughan et al., 2015; Zuo et al., 2015). Recent work has shown that distal lung cells can acquire phenotypic markers of proximal cells under certain conditions *in vitro*. However, whether this same process happens *in vivo* remains unknown (Kathiriya et al., 2022; Louie et al., 2022). Thus, techniques for controlled and simultaneous isolation of both proximal and distal epithelial cells from the same mouse on the basis of their physical location, is critical to further understand their role in disease. In the present study, we develop and validate a new method to simultaneously and more precisely isolate both proximal and distal progenitors from individual mice through the controlled physical separation of the trachea and bronchi from the pulmonary lobes, aided by a three-dimensional (3D) printed surgical guide. We show that the 3D printed lobe divider (3DLD) does not affect viability for either the proximal or distal progenitor cells, and they perform comparably with cells isolated using the classic methods in multiple state-of-the-art *in vitro* assays, including in organoid assays. However, we observe morphological differences between organoids based on the isolation method and identify a small subset of genes related to proximal airway epithelium, which are upregulated only in organoids derived from classically isolated distal cells. Precise separation at the distal part of the bronchi results in isolation of a more transcriptionally homogeneous pool of distal epithelial progenitor cells and minimizes the presence of rare contaminating proximal cells.

RESULTS

The 3D printed lobe divider allows efficient separation among the murine trachea, bronchi, and lung lobes

Classic protocols to isolate epithelial progenitor cells in the distal lung or distal airways (referred to as distal progenitors here) require inflation of the lobes with enzymatic solutions administered through the trachea and bronchi (e.g., dispase), while the isolation of mouse tracheal epithelial cells (referred to hereafter as proximal progenitors) is achieved by incubating the trachea in an enzymatic solution (e.g., Pronase) (Jansing et al., 2018; Lam et al., 2011). To achieve simultaneous isolation of both cell types, the trachea must first serve as a passage to administer the distal dissociation solution, followed by quick ligation of the trachea or bronchi to retain the solutions in the pulmonary lobes for cell isolation. Although this process can be done manually and is often used with tracheal ligation, reproducible placement of surgical knots on the most distal portion of the bronchi requires high surgical skill. We hypothesized that this technical choice might lead to co-isolation of a small amount of upper airway basal cells with distal cell isolation procedures. In order to remove the need for high surgical skill and to allow controllable

and reproducible ligation of the bronchi, we designed a 3D printed lobe divider (Figures 1A–1C). We incorporated a locking mechanism on each side of the device to prevent backflow of the dissociation solution before the surgical knots can be tightened and the trachea and main bronchi resected (Figure 1C; Video S1).

After confirming that our prototype worked, we tested whether its use affected cell isolations for either distal or proximal cells using classically described techniques (Jansing et al., 2018; Lam et al., 2011), as well as in commonly used downstream assays (Figures 1D and 1F). As previous studies involving the isolation of proximal progenitor cells did not first expose these cells to dispase, we first confirmed that a brief and additional enzymatic exposure to dispase in the 3DLD did not negatively affect the ability of isolated proximal cells to attach and proliferate in culture. Cells readily attached and expanded to form confluent monolayers *in vitro* after 6 days (Figures 1D and 1E). Similarly, distal progenitor cells isolated with the 3DLD were viable and formed E-cadherin and pro-SPC$^+$ (surfactant protein C) monolayers after 48 h, in line with previous work (Jansing et al., 2018) (Figures 1F and 1G). Thus, the 3DLD allows simultaneous isolation of progenitor cells from both the proximal and distal lung epithelial compartments from a single animal; these cells proliferate *in vitro* and retain their well-known phenotypic markers.

Expanded proximal progenitors isolated with 3DLD differentiate into various cell types in air-liquid interface cultures and organoid assays

As any alterations in isolation protocols could alter the viability or composition of isolated cells, we next characterized 3DLD-isolated cells more extensively. We first validated whether the use of the 3DLD alters the ability of proximal progenitor cells isolated from single animals to be expanded and differentiated into mature, differentiated airway epithelial subtypes using two well-established and commonly used *in vitro* assays: air-liquid interface (ALI) cultures and organoid formation (Figure 2). After 28 days of air exposure in ALI, proximal progenitors from individual mice developed confluent monolayers and a pseudostratified and polarized epithelium with Krt5$^+$ cells (Figure S1). Histological and scanning electron microscopy confirmed the presence of ciliated cells and α-tubulin$^+$ cells restricted to the apical surface of the monolayers, confirming that the 3DLD does not alter the ability of isolated progenitor cells to expand and form differentiated and polarized monolayers (Figures S1 and 2B). Although morphological and histological assessment confirms the ability for the isolated proximal progenitors to differentiate, the choice of phenotypic markers is somewhat subjective. Thus, we performed bulk RNA sequencing (RNA-seq) of proximal progenitors before and after culture at ALI to comprehensively assess transcriptional changes over time. Principal-component analysis (PCA) of all genes shows clear separation between ALI-differentiated cells and the proximal progenitors (Figure 2C), indicating major transcriptional changes between these two *in vitro* conditions. A total of 9,226 genes were differentially expressed (log fold change > 1, $p < 0.05$; Figure 2D), several of which are known to mark various differentiated proximal epithelial cell populations (Figure 2E; Table S2).

Next, we sought to confirm that expanded proximal progenitors isolated using the 3DLD could form organoids (Figures 2F–2H). 3DLD-isolated proximal progenitors formed organoids with a colony formation efficacy (CFE) of 3.1 %, similar to previous studies (Rock et al., 2009) (Figure 2H). Furthermore, they contained acetylated α-tubulin-positive cells, confirming differentiation in organoid culture (Figure 2I). Thus, the 3DLD isolation procedure did not affect the ability of proximal progenitor cells to be used in at least two common *in vitro* assays.

EpCAM$^+$ distal progenitors differentiate into organoids with different morphologies with 3DLD isolation

Next, we compared whether ligation at the level of the trachea (i.e., classical isolation technique) or at the main stem bronchi (i.e., 3DLD) affects the function of isolated cells in an organoid assay (Figure 3A). Distal progenitors derived from both isolation methods formed organoids with similar CFE to those reported in previous studies (Figure 3B), and the CFE was modestly but significantly higher in the 3DLD organoids (Figure 3C). Furthermore, although both methods produced organoids of various sizes, the 3DLD organoids were smaller in diameter and had a narrower cross-sectional area distribution as compared with those using the classic method, which gave rise to several larger organoids per well (Figures 3D, 3E, and S2).

Next, we sought to characterize the cellular composition of these organoids. Although staining of individual phenotypic markers within organoids can help yield insight into the differentiation state of cells within organoids, it is subjective. scRNA-seq is an alternative approach to characterize the cellular-level transcriptional landscape within organoids (Louie et al., 2022), but is limited in the sequencing depth and some cell types, such as ATI cells, are sensitive to the digestion protocols and droplet-based techniques required for scRNA-seq (Riemondy et al., 2019). Thus, we performed bulk RNA-seq on freshly isolated cell pellets as well as the entire organoid culture well after 14 days to provide a comprehensive overview across the entire transcriptional landscape of the organoids formed using both cell isolation methods. Initial PCA analysis demonstrated that the two isolation methods result in disparate transcriptomic profiles (Figures 3F and S3A), with lower gene variance detected in

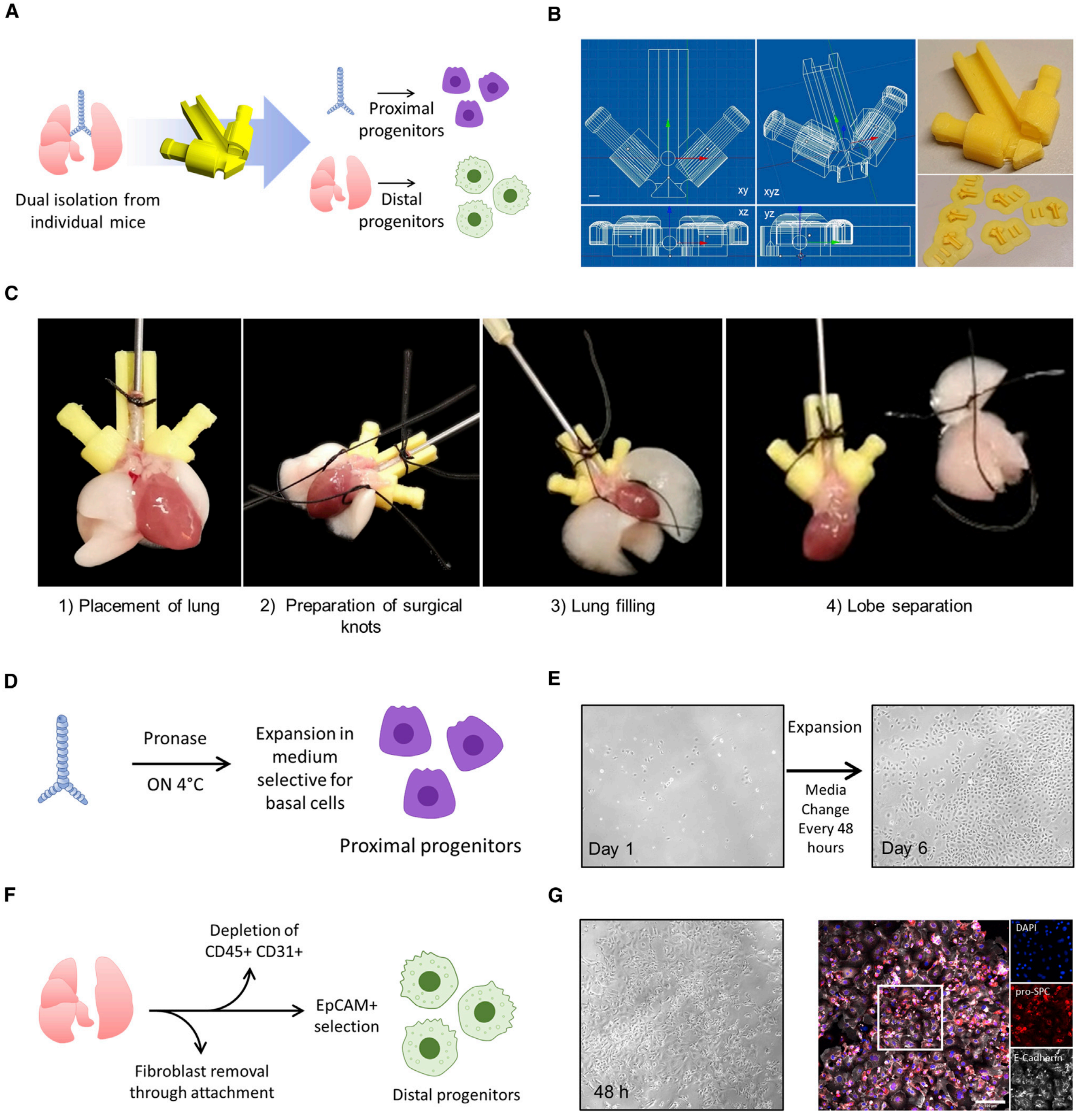

Figure 1. A 3D printed lobe divider (3DLD) allows efficient separation between the murine lung trachea and lobes for isolating proximal and distal progenitor cells from individual mice

(A) Overview of simultaneous isolation of proximal and distal epithelial cells using 3DLD.
(B) Orthogonal and isometric views of 3DLD and final printed product.
(C) Overview of step-by-step 3DLD procedure. See also Video S1.
(D) Isolation and expansion of proximal progenitors.
(E) Representative phase contrast of proximal progenitors after 6 days of expansion. Taken with a 10× objective.
(F) Overview of distal epithelial cell isolation.
(G) Representative immunofluorescent staining of pro-surfactant protein C (pro-SPC) and E-cadherin of distal epithelial cells (taken with a 20× objective).

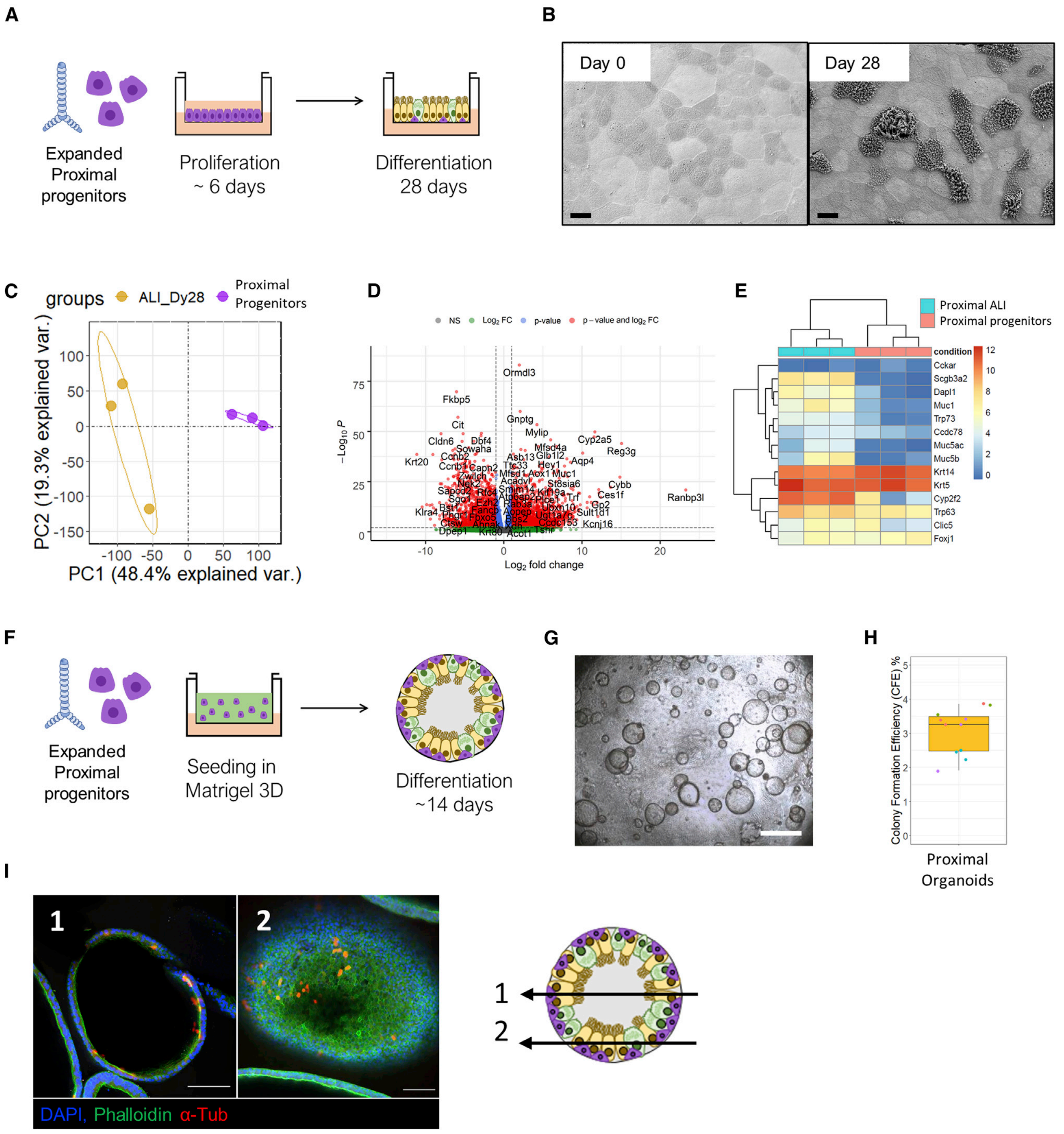

Figure 2. Expanded mouse proximal progenitor epithelial cells isolated with 3DLD differentiate into various cell types in air-liquid interface (ALI) cultures and organoid assays

(A) Schematics for ALI differentiation and organoid formation assay of proximal progenitors. n = 4 individual mice.

(B) Representative scanning electron microscopy (SEM) of ALI culture before lifting to air and 28 days after. Original magnification 1,000× taken at an accelerating voltage of 3.0 kV; scale bar: 10 μm. See also Figure S1.

(C) Principal-component analysis (PCA) of proximal epithelial progenitors before and after 28 days of culture in ALI. Yellow, ALI at day 28; purple, proximal progenitors. n = 3 individual mice.

(legend continued on next page)

cells isolated from individual animals with the 3DLD (Figure S3B). We found 3,270 differentially expressed genes between distal progenitors isolated with the 3DLD or classic method (Figure 3G). Interestingly, genes associated with the proximal epithelium, such as *Krt14*, were among the most differentially expressed as well as genes recently associated with highly proliferative basal cells (*Pbk*) (Succony et al., 2021). Despite differences in organoid size distribution (Figure 3E), organoids formed from both isolation methods resulted in transcriptional profiles which were distinct from their initial starting populations and highly similar to one another (Figure 3F). Nonetheless, small differences in the transcriptional profile could be observed between the organoids derived from the two isolation methods (Figure S3A).

Deconvolution of bulk RNA-seq of distal progenitor pellets reveals higher proportions of contaminating proximal airway cells using a classic isolation protocol

To further explore the transcriptional differences observed in the initial cell pellets (Figures 3F and 3G), we used computational deconvolution of our bulk RNA-seq to gain insights into cellular diversity. Computational deconvolution has been shown to accurately predict experimentally mixed cell types, and is known to work best when the reference scRNA-seq dataset is of the same source as the bulk sample (Dong et al., 2020). Therefore we used a reference dataset which used the classic isolation method (i.e., tracheal ligation) prior to scRNA-seq (Strunz et al., 2020) to computationally deconvolve our bulk RNA-seq data of the cell pellets from both isolation methods using BisqueRNA (Jew et al., 2020) (Figure 4A). To create a scRNA-seq reference set which best matched our experimental setup, we used only untreated cells (i.e., healthy, PBS treated) from the reference dataset and kept the cellular level annotation described in the original reference dataset (Figure 4B). Deconvolution revealed subtle differences among the cell pellets (Figure 4C). We found that distal progenitors isolated with the classic method were predicted to contain a small number of basal cells (see Table S2 for genes used to deconvolve each cell type) in comparison with no predicted basal cells in the 3DLD isolation (Figure 4D). Moreover, a higher proportion of neuroendocrine cells, known to be located mainly in the trachea and upper airways of mice, was also observed in cells isolated with the classic method (Figure 4D). These data indicate that the classical isolation methods that use ligation at the trachea after introducing dissociation buffers may unintentionally isolate contaminating proximal cells.

Next, we explored transcriptional changes occurring within the organoids derived from both isolation methods. We first evaluated changes in phenotypic markers at the bulk RNA-seq level across the different experimental conditions using *Z* scoring of gene lists that define each of the cell clusters from the reference single-cell dataset (Table S2). Interestingly, we noticed a shift towards increased expression of proximal epithelial markers in organoid culture, regardless of the isolation method (Figure 4E), with several of these markers being significantly upregulated (Figure 4F). Next, we compared the transcriptional changes between the organoids and initial cell populations from which they were formed and found that although there were many genes in common that changed during organoid formation, 25 genes were oppositely and significantly regulated (i.e., downregulated in 3DLD relative to 3DLD pellet and upregulated in classic organoids relative to classic pellets) (Figures 4F and 4G). These genes are expressed at various baseline levels and were found predominantly in cells annotated as airway cells in the scRNA-seq reference dataset, such as basal, ciliated, club, and neuroendocrine cells (Figures S3C and S3D). Interestingly, many of these genes are known to be involved in innate immunity, among other biological processes. As these organoids are cultured in the exact same *in vitro* conditions, this indicated that the presence of rare cell populations, because of small differences in the cell isolation method, could have a significant impact on the transcriptional and phenotypic landscape within cultured organoids. Thus, we directly explored whether the addition of freshly isolated proximal cells to distal progenitor cells isolated with the 3DLD at the ratios predicted by deconvolution would cause the same morphological differences observed between classic and 3DLD organoids (Figure 4H). Freshly isolated proximal cells from the same animal, which were isolated with dissociation solutions and selection protocols identical to those used in distal progenitor cell isolations, were added at percentages resembling proportions of “contaminating” proximal cells indicated by deconvolution (1–3%; Figure 4D). Strikingly, we found that the addition of

(D) Volcano plot of differentially expressed genes between expanded proximal progenitors before versus after differentiation in ALI for 28 days. n = 3 individual mice.
(E) Heatmap of select phenotypic markers of differentiated proximal epithelium.
(F) Schematics of organoid formation assay of proximal progenitors.
(G) Representative phase-contrast image of differentiated proximal organoids at day 14. n = 4 individual mice independently; scale bar, 500 μm.
(H) Colony formation efficiency (CFE) of proximal organoids after differentiation for 14 days. n = 4 individual mice; 3 wells quantified per experiment represented by dots colored by mouse.
(I) Immunofluorescence (IF) staining of organoid culture after 14 days. Acetylated α-tubulin (α-tub); scale bar, 100 μm.

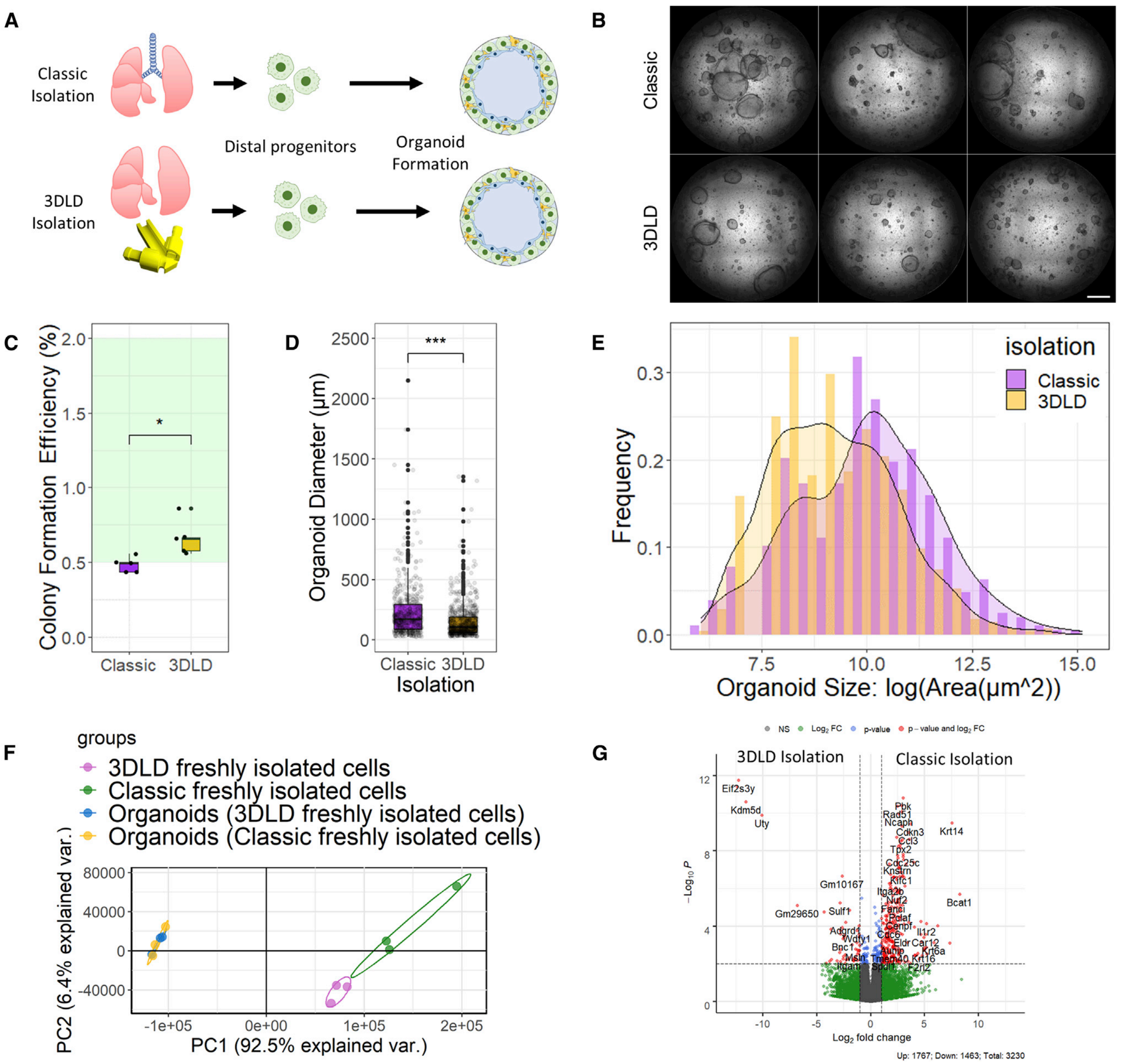

Figure 3. 3DLD isolation alters organoid formation from EpCAM+ distal progenitors

(A) Overview of organoid formation assay using classic or 3DLD isolation methods.

(B) Representative bright-field images of stitched montages of formed organoids after 14 days of culture. n = 3 individual mice; scale bar: 1 mm.

(C) Colony formation efficiency (CFE) from each isolation method. n = 3 individual mice; 2 wells per mouse; *p < 0.05; Mann-Whitney U non-parametric t test.

(D) Organoid diameter; ***p < 0.0001; Mann-Whitney U non-parametric t test.

(E) Frequency size distribution of distal organoids derived from distal progenitors isolated with classic or 3DLD methods. See also Figure S2.

(F) Principal-component analysis (PCA) of distal progenitors isolated with classic or 3DLD methods before and after differentiation in organoid culture. See also Figure S3.

(G) Volcano plot of differentially expressed genes between the cell pellets of 3DLD and classic isolation methods before differentiation.

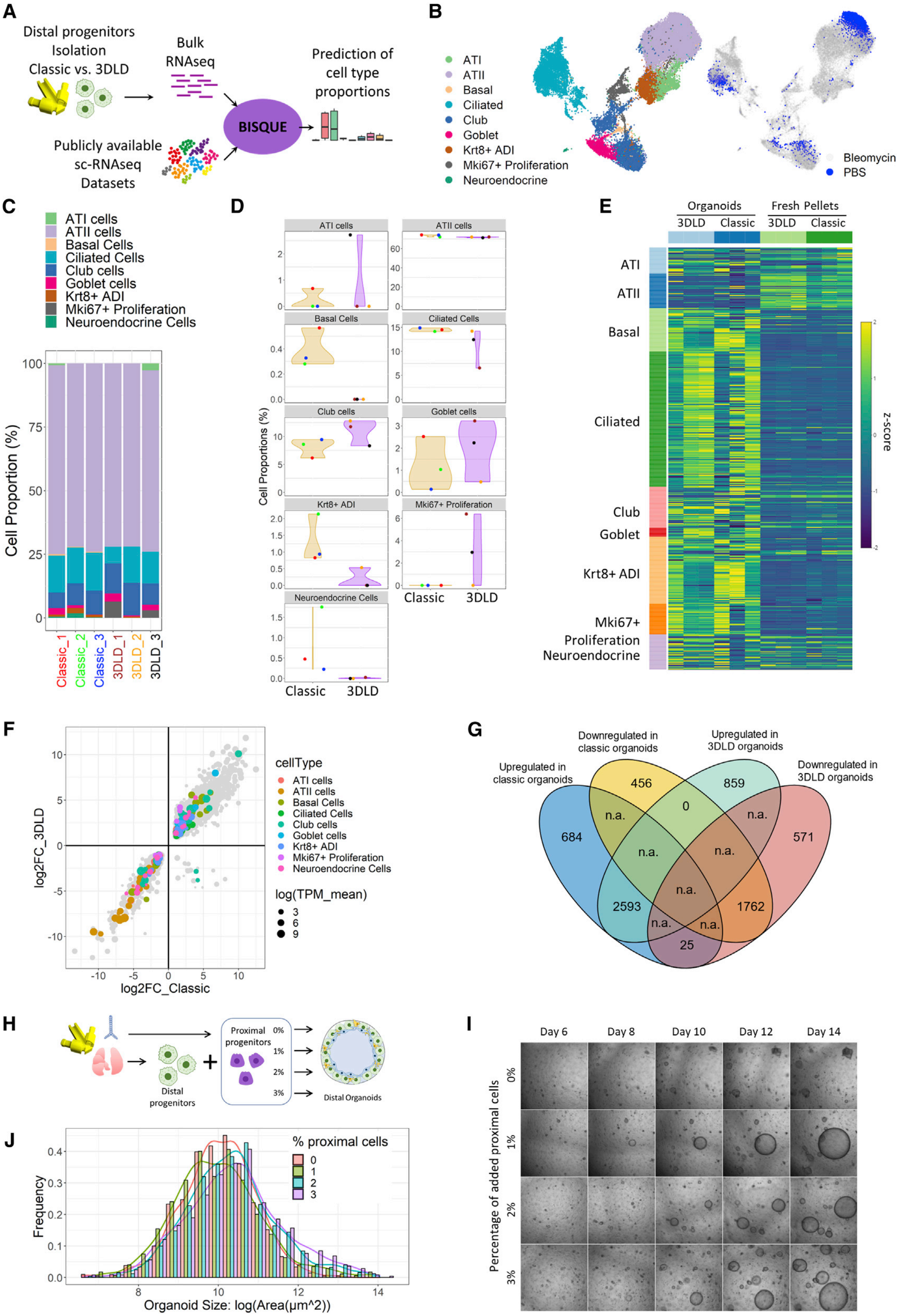

(legend continued on next page)

proximal cells increased organoid size distributions even at the lowest percentage but that this difference was only first evident after 8 days (Figures 4I, 4J, and S4). This provides further support that the presence of rare, contaminating cells derived from the proximal epithelium can affect the trajectory of cultured distal organoids.

DISCUSSION

The lung epithelium is increasingly recognized for its central role in lung homeostasis and disease and a variety of techniques have been employed to study the potential of different epithelial cell progenitors in lung repair and regeneration. Although lineage tracing using reporter animals and scRNA-seq approaches have been invaluable in describing this cellular diversity, the study of isolated and specific lung epithelial cells *in vitro* has been hampered by the lack of surface markers that distinguish the different cells along the respiratory epithelium. Additionally, reporter mice have been previously acknowledged to be “leaky” and to label multiple cell types (Perl et al., 2009). This transcriptional heterogeneity has been confirmed by recent scRNA-seq studies showing that some of the classically described phenotypic markers are expressed in multiple cell types along the entire airway epithelium (Strunz et al., 2020). Additionally, and especially in the case of disease, cells may both lose and/or acquire the phenotypic markers proposed to be used for their isolation. Therefore, the use of reporter mice and even surface markers is not sufficient alone for isolation of well-defined subpopulations along the airway epithelium.

In order to overcome these limitations, the majority of previous approaches for isolating either proximal or distal lung epithelial cells have used physical ligation between the proximal and distal epithelium at the level of the trachea or bronchi and isolated cells through dissociation via enzymatic digestion and selection using (1) *in vitro* culture conditions in the case of proximal epithelial cells to enrich for basal progenitor cells or (2) a combination of negative and positive surface markers for sorting of distal progenitor cells (Corti et al., 1996; Dobbs et al., 1986; Jansing et al., 2018; Lam et al., 2011; Vanderbilt et al., 2015; You et al., 2002). However, both techniques rely on precise surgical techniques to separate the proximal and distal epithelial compartments. Reproducible ligation of the bronchi is surgically challenging in mice and imprecise ligation or variability among laboratories in where and how ligation occurs may therefore inadvertently lead to the co-isolation of undesired cell populations at low frequency. This variability is particularly problematic considering current state-of-the-art techniques for studying lung repair and regeneration, such as organoid assays or scRNA-seq, which seek to identify and characterize the identity and behavior of rare cell populations *ex vivo*. Therefore, and to overcome the aforementioned limitations, we designed and validated the use of a 3D printed guide to reproducibly and reliably isolate both proximal and distal progenitor cells from individual mice for use in multiple state-of-the-art *in vitro* assays such as ALI and organoid culture.

We found that this device allows easy, quick, and reproducible ligation at the level of distal end of the main bronchi, which is otherwise challenging and time consuming in mice. Furthermore, we show that the minor modifications for cell isolation protocols introduced with the 3DLD for dual proximal and distal epithelial cell

Figure 4. Deconvolution of bulk RNA-seq of distal progenitor pellets predicts higher proportions of contaminating proximal airway cells in the classic isolation

(A) Approach for computational deconvolution with BisqueRNA.

(B) Uniform manifold approximation and projection (UMAP) of single-cell dataset re-analyzed on the basis of the original publication (Strunz et al., 2020). Only cells obtained from control mice (treated with PBS) were used in the deconvolution (marked in blue on the right UMAP).

(C and D) Predicted epithelial cell proportion by cell type in the classic or 3DLD isolation method (C) and by individual isolation per mouse (D).

(E) Heatmap of *Z* score of distal progenitors isolated with classic or 3DLD methods before and after differentiation in organoid culture (*Z* score = gene[sample] − gene[mean of all samples]/SD). Genes are selected on the basis of the markers of each cluster in the single-cell dataset (Table S2).

(F) Comparison of the differentially expressed genes between isolated pellets and their corresponding cultured organoids (log_2FC[classic organoids versus pellet] versus log_2FC [3DLD organoids versus pellet]). Marker genes of each cluster are highlighted (Table S2).

(G) Venn diagram of overlapping upregulated and downregulated genes from differential expression analysis of organoids versus pellets. See also Figure S3.

(H) Schematic for manually adding proximal cells isolated using distal epithelial dissociation techniques (i.e., potential contaminating cells) into the distal organoid culture.

(I) Representative, serial bright-field images of distal organoid cultures with the addition of proximal cells isolated from the same animal. n = 1 individual animal. n = 6 experimental replicates; scale bar: 500 μm. Images of the full wells are found in Figure S4.

(J) Frequency size distribution of distal organoids cultured with or without contaminating proximal cells derived from the same animal at day 14. n = 6 experimental replicates.

isolation does not impair the usage of these cells in downstream assays. However, we found that distal epithelial cells freshly isolated with the 3DLD contained lower transcriptional variability compared with those isolated with the classic method, as well as lower amounts of proximal airway cell populations. Thus, our data indicate that the precise separation, aided by 3DLD, of the distal lungs at the main bronchi helps improve reproducibility and precision at which cell isolation occurs.

Heterogeneity in organoid size has been previously observed and, although incompletely understood, has been shown to be altered because of changes in the initial composition of the starting epithelial cell population, type of supporting stromal cell, or the secreted factors in the media (Jain et al., 2015; Shiraishi et al., 2019). In this study, we did not perform additional selection of specific distal lung epithelial progenitors, and thus our organoid culture theoretically contains multiple progenitor populations (Louie et al., 2022). However, we found that introducing contaminating proximal cells into distal organoid culture in similar proportions to those predicted by deconvolution influenced the size of the organoids similarly to what we observed in organoids derived from the classic isolation method. Further studies are needed to understand whether the changes we observe in organoid size between the two isolation methods are due to secretion of paracrine factors altering the distal organoid formation or directly from organoids derived from contaminating cells in the initial cell population.

Nonetheless, most of the transcriptional changes that occurred at the bulk RNA-seq level during organoid formation in Matrigel were similar between the two isolation methods and many of these changes were associated with markers known to define fully differentiated and mature lung epithelial cells, including phenotypic markers characteristic of proximal airways. These changes were evident even in the 3DLD-isolated distal epithelial cells, in which we observed little to no expression of these markers at baseline. The relevance of such changes and whether they can occur *in vivo* is yet to be determined. Matrigel has recently been shown to alter the transcriptional profile of cultured isolated progenitors *ex vivo* at a single-cell level and similar changes are also observed when the same progenitors are transplanted *in vivo* (Louie et al., 2022). However, this points to the increasingly recognized plasticity of epithelial cells observed *in vivo* and *ex vivo*, at least partially governed by the extracellular niche in which these cells reside, which is composed of both matrix and paracrine signaling (Loebel et al., 2021).

Although organoids grown from the 3DLD or classic isolated cells changed similarly relative to their pellets, several genes that are associated with innate immunity were significantly upregulated in organoids formed from the classic isolation, which were downregulated in the 3DLD organoids. These changes were reproducible in all organoids generated from individual mice, indicating that even though the organoids from both isolation methods were cultured in otherwise identical conditions, the composition of the initial cell population significantly affected the trajectory and transcriptional landscape of the final organoid culture. The observation that organoids from classically isolated cells expressed higher transcriptional levels of several markers for innate immunity is particularly interesting as an increasing number of studies have shown a link between innate immunity and lung regeneration (Whitsett and Alenghat, 2015). Therefore, careful characterization of starting cell populations and the isolation methods used to obtain them are critical for interpreting the regenerative capacity of distal and proximal airway epithelial cells and the cues that govern their proliferation and differentiation.

In conclusion, we present a method to simultaneously isolate murine proximal and distal epithelial progenitors from the same mouse lung in a reproducible and inexpensive manner aided by a 3D printed guide. This method allows the evaluation of both the distal and proximal epithelial progenitor populations from a single animal and will allow increased reproducibility across laboratories performing state-of-the-art *ex vivo* analysis for regenerative populations in diverse downstream analyses such as scRNA-seq, organoid assays, or other 3D *in vitro* assays.

EXPERIMENTAL PROCEDURES

Resource availability

Corresponding author

Darcy E. Wagner, PhD (darcy.wagner@med.lu.se).

Materials availability

Ready-to-print STL files for the 3DLD device are available on the GitHub repository (https://github.com/Lung-bioengineering-regeneration-lab/dual_cell_isolation/tree/main/3DLD_design).

Data and code availability

Raw data from the present study are deposited in the European Nucleotide Archive (ENA) under accession number (ENA: E-MTAB-11502). All relevant codes used in this study are available on GitHub (https://github.com/Lung-bioengineering-regeneration-lab/dual_cell_isolation).

Animals

The use of mice was approved by the Local Ethics Committee for Animal Research in Lund, Sweden (Dnr 6436/2017). All animal experiments were conducted in accordance with European Union (2010/63/EU) and local Swedish law. All animals received care according to the Guide for the Care and Use of Laboratory Animals, Eighth Edition (National Academies Press, 2011). C57BL/6N male mice (*Mus musculus*) aged 8–12 weeks were used for all isolations. Mice were housed with same-sex littermates under alternating

light-dark cycles in a humidity- and temperature-controlled room with food and water *ad libitum*. All mice were provided care in accordance with the animal protection and welfare guidelines, and experiments and data are reported in accordance with ARRIVE 2.0 guidelines.

Fused deposition modeling 3D printing

The 3D lobe divider was designed using Blender Software (version 2.8; www.blender.org). The 3D model is available on GitHub (https://github.com/Lung-bioengineering-regeneration-lab/dual_cell_isolation/).

Murine lung explantation and preparation for 3DLD or classic distal isolation methods

All mice were euthanized using a 3:1 ketamine/xylazine mixture, and tracheas were intubated using a modified blunt 19G needle. The lungs were then perfused through the heart with warm PBS (37°C) and removed from the chest cavity. Lungs were immersed in PBS at room temperature (RT) to remove any residual blood. For the 3DLD method, lungs were placed in the 3DLD by placing the trachea in the neck of the device and stabilized with sutures. Loose sutures were placed around both the right and left bronchi in preparation for instillation of dissociation solutions (Figure 1C; Video S1).

3DLD and classic distal cell isolation and organoid formation

For both distal cell isolation procedures, distal dissociation solution (Table S1) was injected through the cannulated trachea. In 3DLD isolations, this was followed by immediate closure of the 3DLD device and closing the sutures surrounding the main bronchi. The lobes were then separated with scissors and placed on ice submerged in distal dissociation solution until proceeding to cell isolation. The trachea was freed from the device and immediately washed and placed in cold F12/AB medium for further processing (Table S1). For the classic isolation, ligation occurred manually on the trachea after injection of the distal dissociation solution and 300 μL 1% agarose (A9414; Sigma-Aldrich). The lungs were placed on ice submerged in distal dissociation solution. Distal progenitor cells for both the classic and 3DLD device were isolated, as previously described (Jansing et al., 2018). Briefly, isolated murine lung lobes were incubated in distal dissociation solution (Table S1) for 45 min at RT. Lobes were then minced using forceps in base/DNase medium (Table S1). Cells were subsequently filtered through 100, 40, and 10 μm meshes sequentially. Fibroblasts and macrophages were removed by allowing cell suspensions to attach on the surface of 10 cm tissue culture Petri dishes for 30 min at 37°C (2 dishes per mouse). Macrophages, monocytes, and endothelial cells were further depleted using the Quadri MACs system (130-091-051; Miltenyi Biotec) using a mixture of anti-CD45 (130-052-301; Miltenyi Biotec) and anti-CD31 (130-097-418; Miltenyi Biotec) microbeads. EpCAM$^+$ (130-105-958; Miltenyi Biotec) cells were selected for organoid formation assays or for cell pellets used in RNA-seq.

Distal organoid formation was done with murine lung fibroblasts (CCL206; American Type Culture Collection [ATCC]) as stromal support cells, as previously described (Costa et al., 2021). Briefly, proliferation of CCL206 cells was inhibited by treatment with 10 μg/mL mitomycin C (M4287; Sigma-Aldrich) for 1 h at 37°C followed by 2 h incubation in CCL206 culture medium. CCL206 cells were lifted and resuspended in Matrigel. A 1:1 ratio of CCL206/Matrigel to EpCAM$^+$ distal progenitor cells (20,000 cells each), totaling 100 μL, was pipetted into 6.5 mm, 0.4 μm Corning Transwells (10482181; Thermo Fisher Scientific) and incubated with distal organoid culture medium (Table S1). Distal organoid medium was changed every 48 h for 14 days.

In experiments with addition of contaminating proximal cells, the 3DLD was used to separate trachea and lobes of the same animal and proximal cells were isolated in the same manner as distal cells following the same incubation steps, times, and selection procedure as the distal lung isolation. Cells were then counted using a hemocytometer with Trypan blue staining and proximal cells were added at various percentages (0%, 1%, 2%, and 3%) to the distal progenitor fraction of the initial organoid culture mixture.

Proximal progenitor isolation and expansion

Proximal progenitors were isolated as previously described with minor modifications (Eenjes et al., 2018; Lam et al., 2011). Connective tissue was removed from the tracheas, and the inner lumen was exposed by cutting the trachea open with surgical scissors. Open tracheas from individual mice were incubated in F12/Pronase overnight at 4°C (Table S1) then washed in F12/AB 3 times to collect digested cells. Cells from each individual mouse were pelleted and treated with 500 μg/mL DNase I (A3778; Saveen & Werner) for 5 min on ice. Cells isolated from individual mice were expanded separately in proximal progenitor expansion medium (Table S1). When cells reached 80%–90% confluence, they were passaged and either seeded submerged, in air-liquid interface culture or in Matrigel for organoid formation, as described in supplemental experimental procedures.

Total RNA isolation and next-generation RNA sequencing

RNA isolation was performed using the RNeasy Micro Kit (74004; Qiagen) including RNase-free DNase I incubation (79254; Qiagen), following the manufacturer's protocol. RNA concentrations were measured using NanoDrop 1000 and Qubit™ RNA HS Assay Kit (Q32852; Thermo Fisher Scientific). RNA quality was evaluated by the Agilent 2100 BioAnalyzer and sequenced using NovaSeq 6000 Sequencing System (20012850; Illumina), as described in supplemental experimental procedures.

Deconvolution of bulk RNA-seq data using publicly available datasets

Bulk RNA-seq data were computationally deconvolved using BisqueRNA to predict the proportions of epithelial cells in the two isolation methods (Jew et al., 2020). The reference single-cell dataset is composed of lung and airway epithelial cells isolated using the classic isolation method for distal lung cells and is available at Gene Expression Omnibus (GEO: GSE141259) (Strunz et al., 2020). Bulk RNA-seq read counts from distal cells of the 3DLD isolation and classic isolation were filtered on the basis of gene expression; only genes with counts in at least 2 of the 3 samples in each of the groups were considered. ExpressionSets were generated from the bulk data and single-cell data on the basis of the original

single-cell reference dataset annotation using Seurat (Butler et al., 2018; Strunz et al., 2020). BisqueRNA deconvolution was done using the function ReferenceBasedDecomposition, with options use.overlap and old.cpm set to false. All code used is available on the GitHub repository (https://github.com/Lung-bioengineering-regeneration-lab/dual_cell_isolation).

Statistical analysis

Statical tests were done using R statistical software and applied where appropriate and are indicated in figure legends. Cutoffs for p values are indicated in each figure.

SUPPLEMENTAL INFORMATION

Supplemental information can be found online at https://doi.org/10.1016/j.stemcr.2022.11.002.

AUTHOR CONTRIBUTIONS

Conceptualization, H.N.A., V.P., L.M., and D.E.W.; Data Curation, H.N.A.; Formal Analysis, H.N.A. and D.E.W.; Funding Acquisition, D.E.W., J.S., and L.M.; Investigation, H.N.A., J.S., I.S., and M.M.; Methodology, H.N.A., J.S., I.S., and D.E.W.; Project Administration, H.N.A., V.P., and D.E.W.; Resources and Software, H.N.A. and D.E.W.; Supervision, J.S. and D.E.W.; Validation and Visualization, H.N.A.; Writing – Original Draft, H.N.A. and D.E.W.; Writing – Review & Editing, all authors.

ACKNOWLEDGMENTS

We thank the Lund University Bioimaging Centre (LBIC) for their assistance in imaging techniques. We thank visiting PhD student Caroline Pereira for insightful conversations and assistance with cell isolation. We thank the Center for Translational Genomics, Lund University, and Clinical Genomics Lund, SciLifeLab, for providing sequencing services. This project is partially funded by the Swedish Research Council Starting Grant (Dnr 2018–02352). The Knut and Alice Wallenberg Foundation is acknowledged for generous support. This project has received funding from the European Research Council (ERC) under the European Union's Horizon 2020 research and innovation program (grant agreement 805361). The light sheet fluorescence microscopy (LSFM) imaging was enabled by resources funded in part by the Crafoord Foundation (Dnr 20200744). Computation/data handling was enabled by resources provided by the Swedish National Infrastructure for Computing (SNIC) at LUNARC partially funded by the Swedish Research Council through grant agreement 2018–05973.

CONFLICT OF INTERESTS

V.P. is a full-time employee and received research funding at AstraZeneca and is a shareholder in AstraZeneca. At the time of contribution, L.M. was a full-time employee and received research funding at AstraZeneca.

Received: May 4, 2022
Revised: November 1, 2022
Accepted: November 1, 2022
Published: December 1, 2022

REFERENCES

Barkauskas, C.E., Chung, M.-I., Fioret, B., Gao, X., Katsura, H., and Hogan, B.L.M. (2017). Lung organoids: current uses and future promise. Development *144*, 986–997. https://doi.org/10.1242/dev.140103.

Barkauskas, C.E., Cronce, M.J., Rackley, C.R., Bowie, E.J., Keene, D.R., Stripp, B.R., Randell, S.H., Noble, P.W., and Hogan, B.L.M. (2013). Type 2 alveolar cells are stem cells in adult lung. J. Clin. Investig. *123*, 3025–3036. https://doi.org/10.1172/JCI68782.

Butler, A., Hoffman, P., Smibert, P., Papalexi, E., and Satija, R. (2018). Integrating single-cell transcriptomic data across different conditions, technologies, and species. Nat. Biotechnol. *36*, 411–420. https://doi.org/10.1038/nbt.4096.

Chapman, H.A., Li, X., Alexander, J.P., Brumwell, A., Lorizio, W., Tan, K., Sonnenberg, A., Wei, Y., and Vu, T.H. (2011). Integrin α6β4 identifies an adult distal lung epithelial population with regenerative potential in mice. J. Clin. Invest. *121*, 2855–2862. https://doi.org/10.1172/jci57673.

Chen, H., Matsumoto, K., Brockway, B.L., Rackley, C.R., Liang, J., Lee, J.-H., Jiang, D., Noble, P.W., Randell, S.H., Kim, C.F., and Stripp, B.R. (2012). Airway epithelial progenitors are region specific and show differential responses to bleomycin-induced lung injury. Stem Cells (Dayton, Ohio) *30*, 1948–1960. https://doi.org/10.1002/stem.1150.

Corti, M., Brody, A.R., and Harrison, J.H. (1996). Isolation and primary culture of murine alveolar type II cells. Am. J. Respir. Cell Mol. Biol. *14*, 309–315. https://doi.org/10.1165/ajrcmb.14.4.8600933.

Costa, R., Wagner, D.E., Doryab, A., De Santis, M.M., Schorpp, K., Rothenaigner, I., Lehmann, M., Baarsma, H.A., Liu, X., Schmid, O., et al. (2021). A drug screen with approved compounds identifies amlexanox as a novel Wnt/β-catenin activator inducing lung epithelial organoid formation. Br. J. Pharmacol. *178*, 4026–4041. https://doi.org/10.1111/bph.15581.

Dobbs, L.G., Gonzalez, R., and Williams, M.C. (1986). An improved method for isolating type II cells in high yield and Purity. Am. Rev. Respir. Dis. *134*, 141–145. https://doi.org/10.1164/arrd.1986.134.1.141.

Dong, M., Thennavan, A., Urrutia, E., Li, Y., Perou, C.M., Zou, F., and Jiang, Y. (2020). SCDC: bulk gene expression deconvolution by multiple single-cell RNA sequencing references. Brief. Bioinform. *22*, 416–427. https://doi.org/10.1093/bib/bbz166.

Eenjes, E., Mertens, T.C.J., Buscop-van Kempen, M.J., van Wijck, Y., Taube, C., Rottier, R.J., and Hiemstra, P.S. (2018). A novel method for expansion and differentiation of mouse tracheal epithelial cells in culture. Sci. Rep. *8*, 7349. https://doi.org/10.1038/s41598-018-25799-6.

Jain, R., Barkauskas, C.E., Takeda, N., Bowie, E.J., Aghajanian, H., Wang, Q., Padmanabhan, A., Manderfield, L.J., Gupta, M., Li, D., et al. (2015). Plasticity of Hopx(+) type I alveolar cells to regenerate type II cells in the lung. Nat. Commun. *6*, 6727. https://doi.org/10.1038/ncomms7727.

Jansing, N.L., McClendon, J., Kage, H., Sunohara, M., Alvarez, J.R., Borok, Z., and Zemans, R.L. (2018). Isolation of rat and mouse alveolar type II epithelial cells. In Lung Innate Immunity and Inflammation: Methods and Protocols, S. Alper and W.J. Janssen,

eds. (Springer New York), pp. 69–82. https://doi.org/10.1007/978-1-4939-8570-8_6.

Jew, B., Alvarez, M., Rahmani, E., Miao, Z., Ko, A., Garske, K.M., Sul, J.H., Pietiläinen, K.H., Pajukanta, P., and Halperin, E. (2020). Accurate estimation of cell composition in bulk expression through robust integration of single-cell information. Nat. Commun. *11*, 1971. https://doi.org/10.1038/s41467-020-15816-6.

Joshi, N., Watanabe, S., Verma, R., Jablonski, R.P., Chen, C.-I., Cheresh, P., Markov, N.S., Reyfman, P.A., McQuattie-Pimentel, A.C., Sichizya, L., et al. (2020). A spatially restricted fibrotic niche in pulmonary fibrosis is sustained by M-CSF/M-CSFR signaling in monocyte-derived alveolar macrophages. Eur. Respir. J. *55*, 1900646. https://doi.org/10.1183/13993003.00646-2019.

Kathiriya, J.J., Wang, C., Zhou, M., Brumwell, A., Cassandras, M., Le Saux, C.J., Cohen, M., Alysandratos, K.-D., Wang, B., Wolters, P., et al. (2022). Human alveolar type 2 epithelium transdifferentiates into metaplastic KRT5+ basal cells. Nat. Cell Biol. *24*, 10–23. https://doi.org/10.1038/s41556-021-00809-4.

Kim, C.F.B., Jackson, E.L., Woolfenden, A.E., Lawrence, S., Babar, I., Vogel, S., Crowley, D., Bronson, R.T., and Jacks, T. (2005). Identification of bronchioalveolar stem cells in normal lung and lung cancer. Cell *121*, 823–835. https://doi.org/10.1016/j.cell.2005.03.032.

Lam, H.C., Choi, A.M., and Ryter, S.W. (2011). Isolation of mouse respiratory epithelial cells and exposure to experimental cigarette smoke at air liquid interface. J. Vis. Exp. *2513*, 2513. https://doi.org/10.3791/2513.

Loebel, C., Weiner, A.I., Katzen, J.B., Morley, M.P., Bala, V., Cardenas-Diaz, F.L., Davidson, M.D., Shiraishi, K., Basil, M.C., Ochs, M., et al. (2021). Microstructured hydrogels to guide self-assembly and function of lung alveolospheres. Preprint at bioRxiv. https://doi.org/10.1101/2021.08.30.457534.

Louie, S.M., Moye, A.L., Wong, I.G., Lu, E., Shehaj, A., Garcia-de-Alba, C., Ararat, E., Raby, B.A., Lu, B., Paschini, M., et al. (2022). Progenitor potential of lung epithelial organoid cells in a transplantation model. Cell Rep. *39*, 110662. https://doi.org/10.1016/j.celrep.2022.110662.

McQualter, J.L., Yuen, K., Williams, B., and Bertoncello, I. (2010). Evidence of an epithelial stem/progenitor cell hierarchy in the adult mouse lung. Proc. Natl. Acad. Sci. USA *107*, 1414–1419. https://doi.org/10.1073/pnas.0909207107.

Messier, E.M., Mason, R.J., and Kosmider, B. (2012). Efficient and rapid isolation and purification of mouse alveolar type II epithelial cells. Exp. Lung Res. *38*, 363–373. https://doi.org/10.3109/01902148.2012.713077.

Montoro, D.T., Haber, A.L., Biton, M., Vinarsky, V., Lin, B., Birket, S.E., Yuan, F., Chen, S., Leung, H.M., Villoria, J., et al. (2018). A revised airway epithelial hierarchy includes CFTR-expressing ionocytes. Nature *560*, 319–324. https://doi.org/10.1038/s41586-018-0393-7.

Perl, A.-K., Zhang, L., and Whitsett, J.A. (2009). Conditional expression of genes in the respiratory epithelium in transgenic mice. Am. J. Respir. Cell Mol. Biol. *40*, 1–3. https://doi.org/10.1165/rcmb.2008-0011ED.

Rawlins, E.L. (2008). Lung epithelial progenitor cells. Proc. Am. Thorac. Soc. *5*, 675–681. https://doi.org/10.1513/pats.200801-006AW.

Rawlins, E.L., Okubo, T., Xue, Y., Brass, D.M., Auten, R.L., Hasegawa, H., Wang, F., and Hogan, B.L.M. (2009). The role of Scgb1a1+ Clara cells in the long-term maintenance and repair of lung airway, but not alveolar, epithelium. Cell Stem Cell *4*, 525–534. https://doi.org/10.1016/j.stem.2009.04.002.

Reynolds, S.D., Giangreco, A., Power, J.H., and Stripp, B.R. (2000). Neuroepithelial bodies of pulmonary airways serve as a reservoir of progenitor cells capable of epithelial regeneration. Am. J. Pathol. *156*, 269–278. https://doi.org/10.1016/S0002-9440(10)64727-X.

Riemondy, K.A., Jansing, N.L., Jiang, P., Redente, E.F., Gillen, A.E., Fu, R., Miller, A.J., Spence, J.R., Gerber, A.N., Hesselberth, J.R., and Zemans, R.L. (2019). Single cell RNA sequencing identifies TGFβ as a key regenerative cue following LPS-induced lung injury. JCI insight *5*, e123637. https://doi.org/10.1172/jci.insight.123637.

Rock, J.R., Onaitis, M.W., Rawlins, E.L., Lu, Y., Clark, C.P., Xue, Y., Randell, S.H., and Hogan, B.L.M. (2009). Basal cells as stem cells of the mouse trachea and human airway epithelium. Proc. Natl. Acad. Sci. *106*, 12771–12775. https://doi.org/10.1073/pnas.0906850106.

Rock, J.R., Randell, S.H., and Hogan, B.L.M. (2010). Airway basal stem cells: a perspective on their roles in epithelial homeostasis and remodeling. Dis. Model. Mech. *3*, 545–556. https://doi.org/10.1242/dmm.006031.

Shiraishi, K., Shichino, S., Ueha, S., Nakajima, T., Hashimoto, S., Yamazaki, S., and Matsushima, K. (2019). Mesenchymal-epithelial interactome analysis reveals essential factors required for fibroblast-free alveolosphere formation. iScience *11*, 318–333. https://doi.org/10.1016/j.isci.2018.12.022.

Sikkema, L., Strobl, D., Zappia, L., Madissoon, E., Markov, N., Zaragosi, L., Ansari, M., Arguel, M., Apperloo, L., Bécavin, C., et al. (2022). An integrated cell atlas of the human lung in health and disease. Preprint at bioRxiv. https://doi.org/10.1101/2022.03.10.483747.

Strunz, M., Simon, L.M., Ansari, M., Kathiriya, J.J., Angelidis, I., Mayr, C.H., Tsidiridis, G., Lange, M., Mattner, L.F., Yee, M., et al. (2020). Alveolar regeneration through a Krt8+ transitional stem cell state that persists in human lung fibrosis. Nat. Commun. *11*, 3559. https://doi.org/10.1038/s41467-020-17358-3.

Succony, L., Gómez-López, S., Pennycuick, A., Alhendi, A.S.N., Davies, D., Clarke, S.E., Gowers, K.H.C., Wright, N.A., Jensen, K.B., and Janes, S.M. (2021). em>Lrig1 expression identifies airway basal cells with high proliferative capacity and restricts lung squamous cell carcinoma growth. Eur. Respir. J., 2000816. https://doi.org/10.1183/13993003.00816-2020.

Tata, P.R., and Rajagopal, J. (2017). Plasticity in the lung: making and breaking cell identity. Development *144*, 755–766. https://doi.org/10.1242/dev.143784.

Vanderbilt, J.N., Gonzalez, R.F., Allen, L., Gillespie, A., Leaffer, D., Dean, W.B., Chapin, C., and Dobbs, L.G. (2015). High-efficiency type II cell-enhanced green fluorescent protein expression facilitates

cellular identification, tracking, and isolation. Am. J. Respir. Cell Mol. Biol. *53*, 14–21. https://doi.org/10.1165/rcmb.2014-0348MA.

Vaughan, A.E., Brumwell, A.N., Xi, Y., Gotts, J.E., Brownfield, D.G., Treutlein, B., Tan, K., Tan, V., Liu, F.C., Looney, M.R., et al. (2015). Lineage-negative progenitors mobilize to regenerate lung epithelium after major injury. Nature *517*, 621–625. https://doi.org/10.1038/nature14112.

Volckaert, T., and De Langhe, S. (2014). Lung epithelial stem cells and their niches: Fgf10 takes center stage. Fibrogenesis Tissue Repair *7*, 8. https://doi.org/10.1186/1755-1536-7-8.

Whitsett, J.A., and Alenghat, T. (2015). Respiratory epithelial cells orchestrate pulmonary innate immunity. Nat. Immunol. *16*, 27–35. https://doi.org/10.1038/ni.3045.

You, Y., Richer, E.J., Huang, T., and Brody, S.L. (2002). Growth and differentiation of mouse tracheal epithelial cells: selection of a proliferative population. Am. J. Physiol. Lung Cell Mol. Physiol. *283*, L1315–L1321. https://doi.org/10.1152/ajplung.00169.

Zuo, W., Zhang, T., Wu, D.Z., Guan, S.P., Liew, A.-A., Yamamoto, Y., Wang, X., Lim, S.J., Vincent, M., Lessard, M., et al. (2015). p63+Krt5+ distal airway stem cells are essential for lung regeneration. Nature *517*, 616–620. https://doi.org/10.1038/nature13903.

Stem Cell Reports, Volume *17*

Supplemental Information

Simultaneous isolation of proximal and distal lung progenitor cells from individual mice using a 3D printed guide reduces proximal cell contamination of distal lung epithelial cell isolations

Hani N. Alsafadi, John Stegmayr, Victoria Ptasinski, Iran Silva, Margareta Mittendorfer, Lynne A. Murray, and Darcy E. Wagner

Figure S1. Differentiation of 3DLD isolated proximal epithelial cells into a pseudostratified epithelium with normal apical-basal polarity. (A) Hematoxylin and eosin staining and bright field imaging of differentiated proximal epithelial cells cultured in ALI at day 28 confirming the presence of a pseudostratified monolayer. Imaged at 40X magnification (B) Immunofluorescence staining of cytokeratin5 (Krt5, apical layer), alpha-tubulin (α-Tub, cilia in the apical layer). Related to Figure 2. Scale bar: 50 µm. (C) Scanning electron microscopy of differentiated mouse proximal progenitor cells after 28 days of air liquid interface culture. Related to Figure 2.

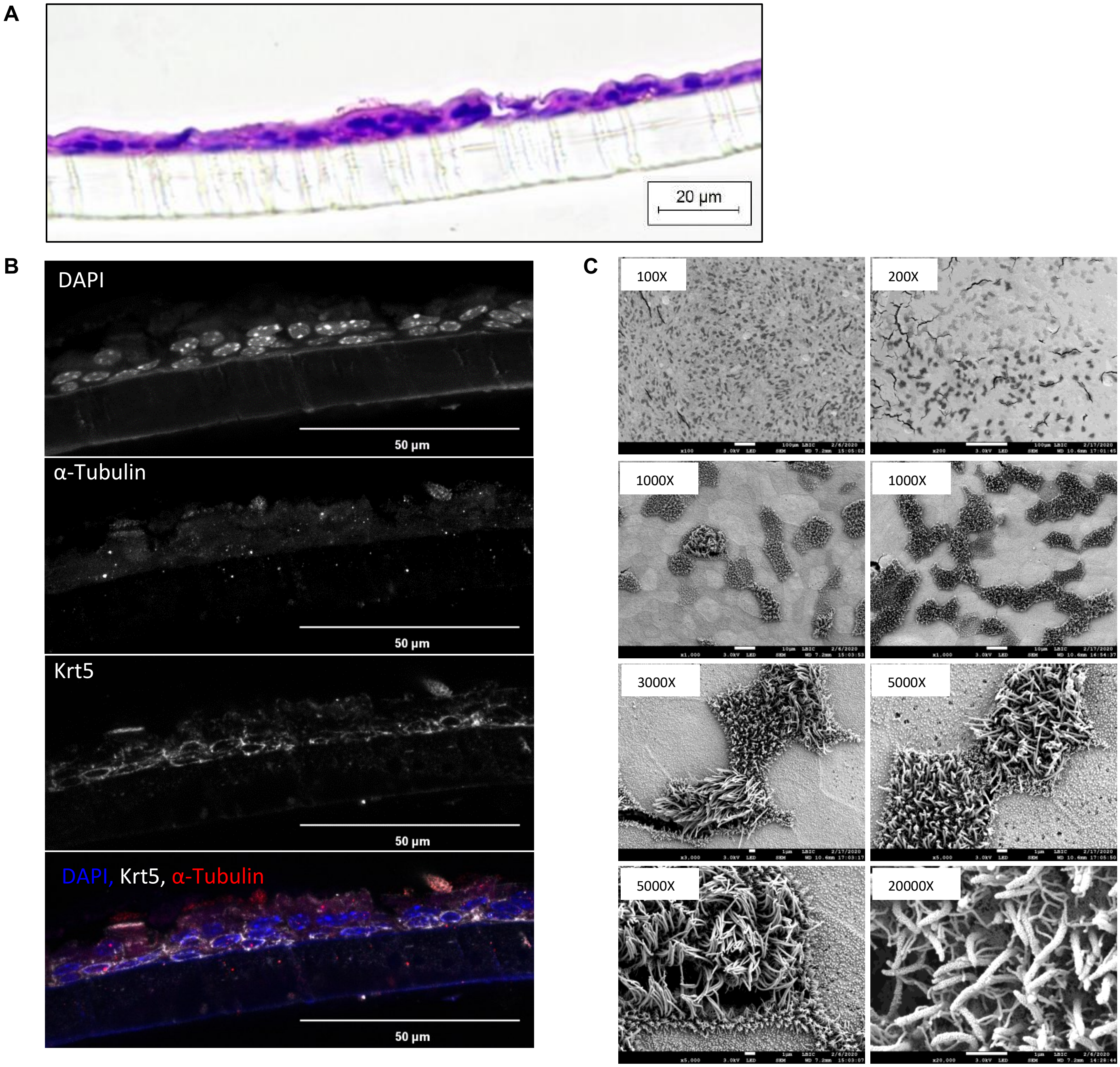

Figure S2. Lightsheet fluorescent microscopy imaging of organoids grown from distal progenitors isolated using either the classic or 3DLD methods. Related to Figure 3. (A) Representative 2D images of different regions of interest (ROI) obtained from the z-stacks to display the nuclear (DAPI) staining of different size organoids. Scale bar 200um. (B) Maximum intensity projection of the DAPI staining of organoids followed by segmentation of each organoid using virtual reality (VR) software SyGlass. These images were obtained at 0.4μm z-resolution which would not be otherwise possible with another imaging modality without photobleaching the sample.

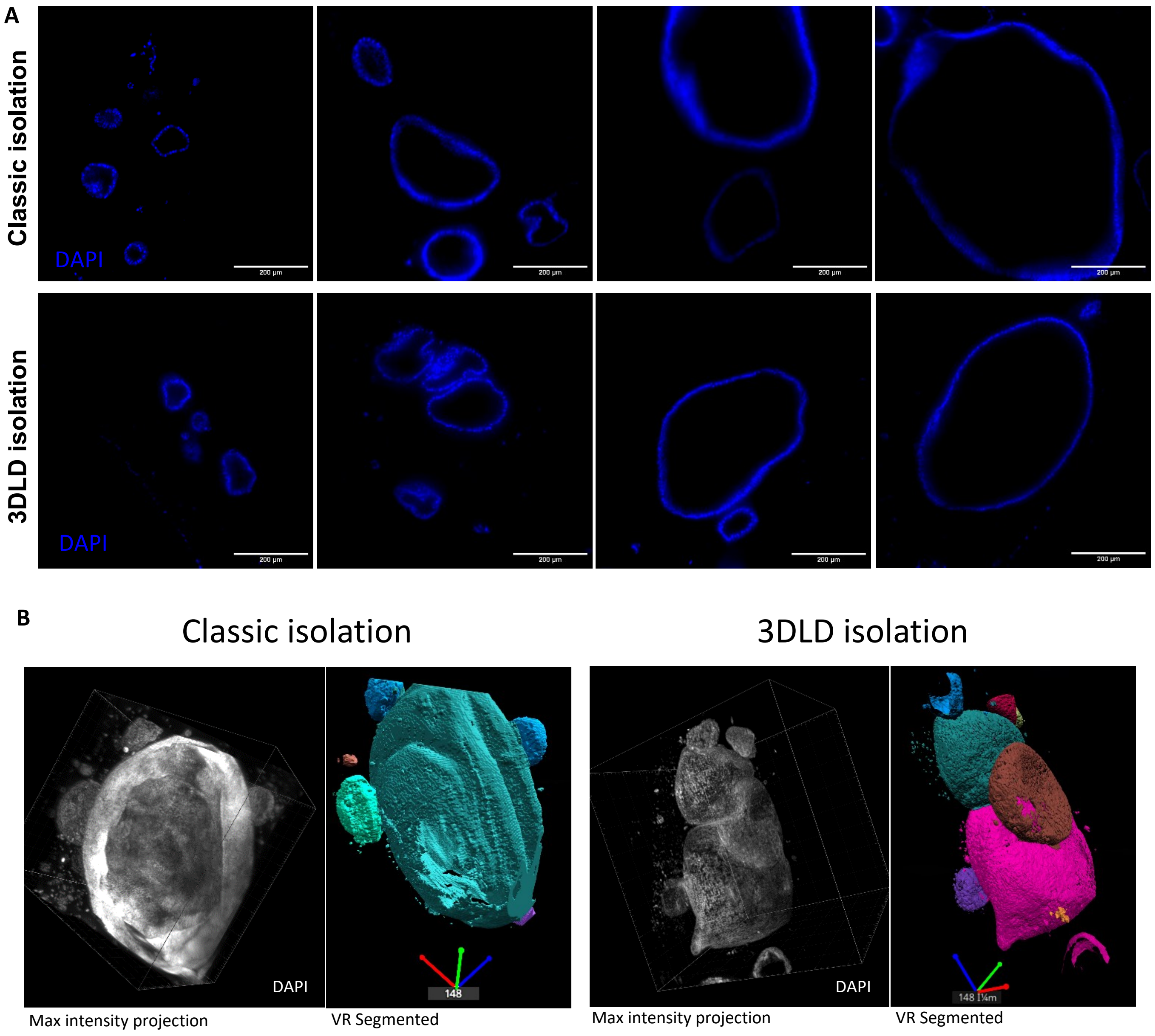

Figure S3. RNAseq analysis of freshly isolated distal cells with the classic or 3DLD isolation before and after organoid culture. Related to Figure3 and Figure 4. (A) Principal component analysis (PCA) of all TPM values for all samples using principal components (PCs) PC1vsPC3. (B) Direct comparison of the relative variance for each gene among the freshly isolated cells using the classic or 3DLD methods. (C) Genes that are oppositely and significantly regulated along the expression trajectory of organoid culture (i.e. downregulated in 3DLD relative to 3DLD pellet and upregulated in classic organoids relative to classic pellets) shown in transcripts per million (TPM). (D) FeaturePlots projected on publicly available single cell Dataset (GSE141259) of epithelial cells isolated in a similar manner to the classic isolation. Scaled data is used to generate the featureplots using the Seurat package on R.

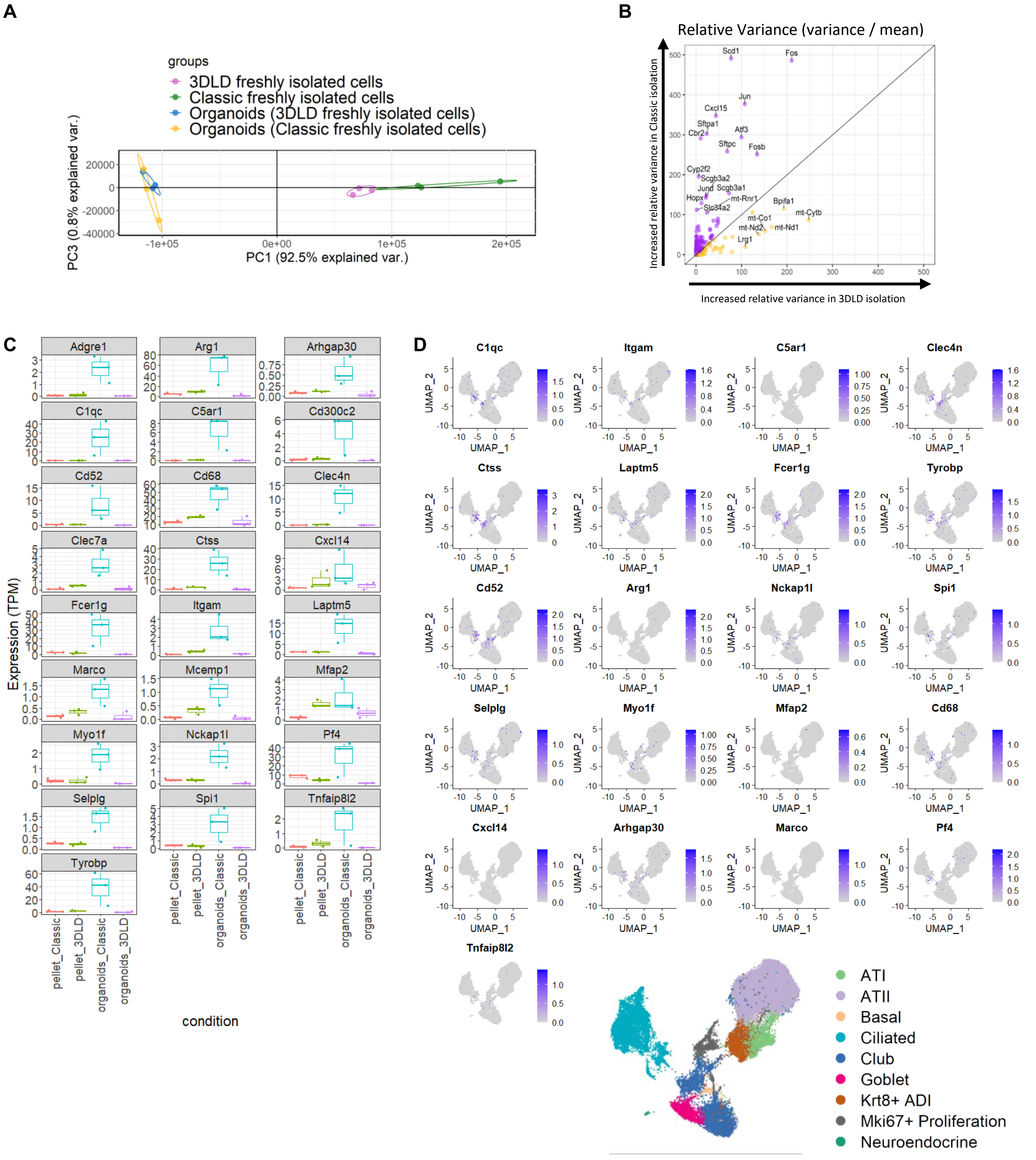

Figure S4. Distal organoids derived from distal progenitors isolated with 3DLD with the addition of proximal cells from the same mouse at concentrations that could represent contamination of proximal cells in classic isolation. Related to Figure 4. (A) Full well brightfield images. Images of full wells were rotated to match the direction on which the organoids were cultured. Image transformation was done using an ImageJ tool (Interactive Well Transformer). Scale bar: 1 mm. (B) Percentage of organoids in each size group divided based on the 4 quantiles of the size distribution observed in Figure 4J.

A

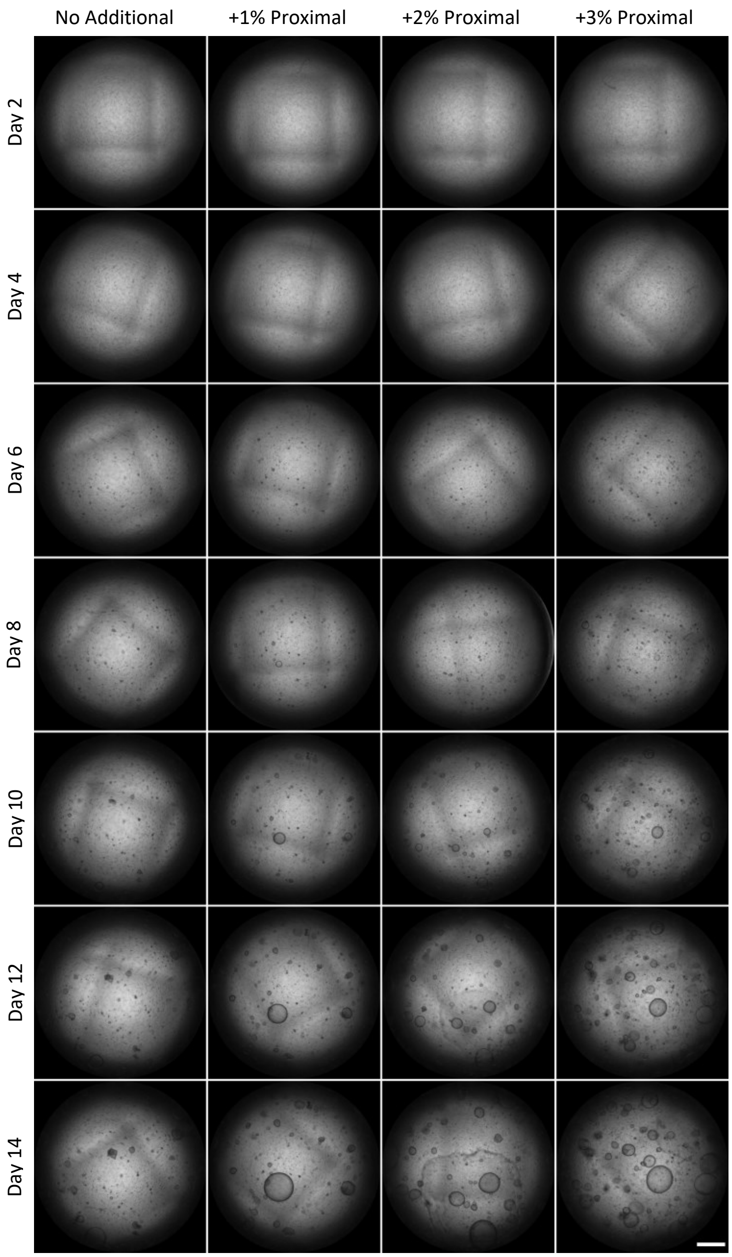

B

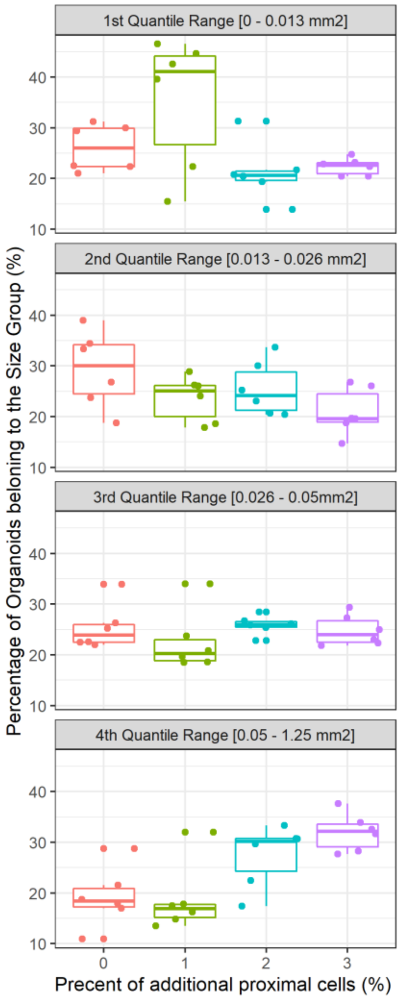

Table S1: Composition of all buffers and solutions. (Table is provided as an excel file)

Table S2: Top genes per cell type used in deconvolution. Related to figure S3 and figure 4. Gene lists are obtained using the function FindAllMarkers() on Seurat in GSE141259 Genes with logFC of higher than 0.5 displayed in this table.

Cell type	Genes
ATI cells	Ager, Akap5, Anxa1, Anxa3, App, Aqp5, Cldn18, Clic3, Clic5, Clu, Cryab, Ctnna1, Ctsh, Emp2, Icam1, Igfbp7, Itgb6, Lgals3, Msn, Myl12a, Myl6, Ndnf, Phldb2, Serpinb6b, Serpinb9, Sparc, Timp3, Tpm1, Tpm3, Vamp8, Vegfa,
ATII cells	Abca3, Acot7, Acsl4, Atp5e, Atrx, Cd36, Ctsc, Cxcl15, Dbi, Dram1, Egfl6, Elovl1, Fabp5, Fasn, H2-K1, Hc, Ifi27l2a, Il33, Lamp3, Lcn2, Lpcat1, Lrg1, Lrp2, Ly6e, Lyz1, Lyz2, Malat1, Napsa, Npc2, Ptgs1, Rgcc, Rnase4, S100g, Scd1, Sfta2, Sftpa1, Sftpb, Sftpc, Sftpd, Slc34a2, Slpi, Tgoln1,
Basal Cells	Abi3bp, Apoe, Aqp3, Aqp4, Bpifa1, Dapl1, Dcn, Dst, Eef1a1, Eef1b2, Egr1, Ehf, Emb, Epas1, F3, Fxyd3, Glul, Gsta4, Gstm2, Gsto1, Jun, Krt15, Krt19, Krt5, Ltf, mt-Co1, Pabpc1, Rpl13a, Rpl14, Rpl18a, Rpl21, Rpl22, Rpl28, Rpl3, Rpl31, Rpl37a, Rpl5, Rpl8, Rplp2, Rps12, Rps16, Rps2, Rps3, Rps4x, Rps8, Rps9, Rpsa, S100a6, Tgm2, Tmem176a, Tmem176b, Tpt1, Zfp36l1,
Ciliated Cells	Acsl3, Aebp1, Agr3, Ak7, Akap14, Akap9, Aldh1a1, Aldh3b1, Anxa2, Anxa8, Aoc1, Arhgap18, Arl3, Ascc1, Atpif1, AU040972, B9d1, BC051019, Bphl, Calm1, Calml4, Capsl, Ccdc113, Ccdc146, Ccdc153, Ccdc17, Ccdc173, Ccdc181, Ccdc30, Ccdc39, Ccdc78, Cd24a, Cd55, Cdhr3, Cdhr4, Cep290, Cetn2, Cetn4, Chchd10, Chchd2, Chchd6, Ckb, Crip2, Cspp1, Csrp2, Cxcl17, Cyp2s1, Cyp4b1, Ddx5, Dnah12, Dnah3, Dnah5, Dnah6, Dnali1, Dpy30, Drc1, Dusp14, Dynll1, Dynlrb2, Efcab1, Efcab10, Eif1, Elof1, Enkur, Erich2, Ezr, Fam161a, Fam183b, Fam216b, Fhad1, Foxj1, Fth1, Gm1673, Gm973, Gsn, Gstm1, H3f3b, Hes6, Hint1, Hsp90aa1, Hspa4l, Hsph1, Ifitm1, Ift43, Ift74, Ift81, Ift88, Igfbp5, Iqca, Iqcg, Itm2b, Kif21a, Lgals3bp, Lrrc23, Lrrc51, Lrrc6, Lrrc71, Lrriq1, Map1b, Mcee, Meig1, Mlf1, Mns1, Mrps17, Mt1, Mycbp, Nek5, Nme5, Nudc, Nudt4, Odf3b, Osbpl6, Pcp4l1, Pebp1, Pifo, Plcb3, Pltp, Ppia, Ppil6, Ppp1r36, Prdx5, Ptges3, Rfk, Riiad1, Rsph1, Rsph4a, Rsph9, S100a1, S100a11, Sec14l3, Slc25a5, Smim5, Sntn, Spa17, Spag17, Spag6, Spef2, Srsf10, Stk33, Stmnd1, Tcea3, Tekt1, Tm4sf1, Tmem107, Tmem212, Tomm7, Tppp3, Traf3ip1, Trp53bp2, Tspan1, Tuba1a, Tubb4b, Txnip, Vpreb3, Wdr66,
Club cells	Alas1, AW112010, B430010I23Rik, Bpifb1, Bst2, C1qb, Cbr2, Ccl17, Ccl6, Cd52, Cd74, Cldn10, Cp, Crip1, Cst3, Ctss, Cyp2f2, Fau, Fmo2, Fmo3, Gsta3, H2-Aa, H2-Ab1, H2-Eb1, Ifit1, Ifit2, Ifit3, Ifitm3, Iigp1, Isg15, Mgst1, mt-Cytb, mt-Nd2, Phf11d, Pigr, Prdx6, Psap, Reg3g, Rsad2, S100a4, Scgb1a1, Scgb3a1, Selenbp1, Spp1, Stat1, Tff2, Tmsb4x, Trf, Vim, Wfdc2,
Goblet cells	Adh7, Agr2, Chad, Gp2, Hp, Lypd2, Muc5b, Qsox1, Retnla, Scgb3a2, Sult1d1,
Krt8+ ADI	Anxa5, Areg, Arpc2, B2m, Calm2, Ccng1, Cd81, Cdkn1a, Cldn4, Cstb, Cystm1, Dstn, Epcam, Esd, Ftl1, Gas5, Gdf15, H2-D1, Hbegf, Hspb1, Ifrd1, Itgb1, Krt18, Krt7, Krt8, Lmo7, Ly6a, Mt2, Myl12b, Nupr1, Pkm, Rpl11, Rpl12, Rpl13, Rpl15, Rpl17, Rpl19, Rpl23, Rpl23a, Rpl26, Rpl27, Rpl27a, Rpl32, Rpl34, Rpl35a, Rpl37, Rpl38, Rpl4, Rpl6, Rpl9, Rplp0, Rplp1, Rps11, Rps13, Rps14, Rps15, Rps15a, Rps17, Rps18, Rps19, Rps20, Rps23, Rps24, Rps25, Rps26, Rps27, Rps27a, Rps27l, Rps29, Rps3a1, Rps5, Rps6, Rps7, S100a10, Sfn, Sprr1a, Sqstm1, Tnfrsf12a, Tnip3, Tpm4, Txn1, Ubb,
Mki67+ Proliferation	Actb, Anln, Anp32b, Arl6ip1, Birc5, Cenpe, Cenpf, Dek, Hmgb1, Hmgb2, Hmgn2, Hmmr, Hnrnpa2b1, Hnrnpa3, Hnrnpab, Hsp90b1, Kif23, Lig1, Mki67, Ncl, Npm1, Pcna, Prc1, Ptma, Ranbp1, Rpl35, Serbp1, Smc2, Smc4, Spc24, Stmn1, Top2a, Tpx2, Tuba1b, Tubb5, Ube2c, Ybx1,
Neuroendocrine Cells	Anp32a, Arl4a, Ascl1, Calca, Car8, Cbx5, Ccnd2, Cd9, Celf4, Chga, Chgb, Col8a1, Cplx2, Ddc, F5, Fos, Gadd45b, Gnas, Hsp90ab1, Hspa5, Id4, Igf2, Itm2c, Meg3, Meis2, Nktr, Nnat, Ntn4, Pam, Pcsk1, Peg3, Piezo2, Pnmal2, Ptn, Ptprn, Resp18, Rgs2, Scg2, Scg5, Spock3, Syt7, Tspan13, Ttc3,

Supplemental experimental procedures

Fused Deposition Modelling (FDM) 3D printing parameters

Standard Tessellation Language (STL) models were sliced using Ultimaker Cura software (v4.0, Ultimaker) at a resolution of 0.1mm. Sliced models were printed using an Ultimaker 3 printer using PLA filaments (Creative Tools, www.creativetools.se). The 3D printer's nozzle diameter was 0.4 mm, infill printing temperature was set at 205 °C, and heated-bed temperature: 60 °C. Prints were sterilized by incubation in 70 % ethanol for a minimum of 1 hour.

Organoid Counting and size distribution

Bright field images of all organoid cultures were captured using a wide-field camera in a Cytation5 Multimodal imager/plate reader with a 4X objective (AH Diagnostics). Each Transwell culture was imaged as a mosaic with z-stacking of 3 planes across the Transwell culture with 219 µm between each plane. All organoids were visible in each z-plane at different focal planes, however, a focal stacking of all 3 planes resulted in a focused 2D image for all organoids making it suitable for analysis. The selection tool in FIJI was used to quantify the number of individual organoids and their size distribution based on area. Moreover, the brightfield images for the full wells were cropped and rotated to match the direction of the well during organoid culture using a custom interactive macro, which can be found on the github repository (https://github.com/Lung-bioengineering-regeneration-lab/IWT) (Alsafadi, 2022).

Air liquid interface (ALI) and organoid formation assay of proximal progenitors

ALI cultures of proximal progenitors were done as previously described (Eenjes et al., 2018). Briefly, expanded cells from individual tracheas were seeded on 6.5 mm 0.4 µm Transwell inserts (10482181, Fisher Scientific) and submerged in proximal progenitors' proliferation medium until confluence (typically day 7) (Table S1). Cells were lifted to air after reaching confluence by only placing liquid on the basal side of the Transwell and proximal differentiation medium was changed every 48 hours for 28 days. Proximal organoids were grown as previously described (Rock et al., 2009; You et al., 2002). Expanded cells from individual tracheas were seeded in 100 µl Matrigel (11543550, Fisher Scientific, Sweden) at 10 000 cells/insert and were incubated in MTEC organoid medium for 14 days with media changes every 48 hours.

Scanning Electron Microscopy (SEM)

Differentiated proximal lung epithelial cells in ALI were fixed in 2.5 % Glutaraldehyde (G5882, Sigma-Aldrich) in PBS at 4 °C overnight and washed in PBS. Fixed samples were dehydrated in an ethanol series 50, 70, 80, 90, 100, 100 % for 10 minutes each at RT while they remained in the insert to avoid warping of the ALI membrane. Critical point drying (CPD) was done using K850 critical point dryer (K80, Quorum, UK). Dried ALI membranes were separated from the inserts and mounted face-up on SEM mounts (PELCO Tabs™, Carbon Conductive Tabs, 25 mm OD, Tedpella) and were sputter coated with platinum/palladium 80/20 at a thickness of 10 nm. Images were acquired using a Jeol JSM-7800F SEM microscope.

Histology and Immunofluorescence (IF).

For histological assessment, samples were fixed in 10 % neutral-buffered formalin at 4 °C overnight and were processed using a xylene-free approach, using graded ethanol series and isopropanol (both Fisher Scientific, UK) before paraffin embedding (Histolab, Sweden). 4 µm wide sections were sliced and placed on microscope slides (Thermo Scientific, Germany) for staining. Deparaffinized sections was followed by staining with hematoxylin and eosin (Merck Millipore, Germany) followed by dehydration in consecutively graded ethanol series. Dried sections were mounted with Pertex (Histolab, Sweden). Antigen retrieval was performed with 10 mM citrate buffer (pH 6.0) in a pressure cooker for 20 minutes at 105 °C. For IF of paraffin sections, blocking was done for 1 hour at RT using 1X PBS / 5 % goat serum / 0.3 % Triton™ X-100, primary antibodies (AB) (Table S1) were diluted in AB dilution buffer consisting of 1X PBS / 1 % BSA / 0.3 % Triton™ X-100 and incubated at 4 °C overnight. The sections were incubated with phalloidin (1:100, A12379, Life Technologies) and the secondary antibodies at RT for 1 hour followed by 4′,6-diamidino-2-phenylindole (DAPI) staining at 5 µg/ml for 10 minutes at RT.

Light-sheet sample preparation and imaging of distal lung organoids

Distal organoid cultures were fixed in 10 % formalin (neutral-buffered and methanol-free) for 2 hours at RT and washed in PBS. Sample preparation and immunolabeling were done using the iDISCO protocol with some modifications (Renier et al., 2014). First, samples were dehydrated in methanol / PBS series (20, 40, 60, 80, 100, 100 % methanol in PBS) for 15 minutes for each step at RT. Delipidation was performed by incubating the samples in a mixture of dichloromethane (DCM, Sigma-Aldrich 270997) and methanol (66 % DCM / 33 % methanol) for 3 hours at RT. Samples were then rehydrated using a methanol / PBS series in the opposite order for 15 minutes each at RT. Samples were then incubated in permeabilization solution (20 % DMSO / 0.16 % Triton-X100 / 2.3 % glycine in PBS) overnight at 37 °C followed by incubation in blocking solution (6 % goat serum / 10 % DMSO / 0.168 % Triton-X100 in PBS) overnight at 37 °C. Primary antibodies were incubated overnight at 4 °C followed by washing in PBS 3 times while shaking for 1 hour each time. Secondary antibodies were incubated for 2 hours at RT and DAPI was incubated for 15 minutes at RT followed by washing in PBS 3 times while shaking for 1 hour each time. After immunolabeling, organoid samples were taken out of the inserts and embedded in 1 % low-gelling temperature agarose (Sigma Aldrich A9414) in milliQ water followed by dehydration using the methanol / PBS series for 1 hour in each step at RT. Samples were then incubated in DCM / methanol (66 % / 33 %) for 1 hour followed by 2 changes of 100 % DCM for 15 minutes each while shaking. Reflective index (RI) matching of the processed samples was

done by incubating the sample in ethyl cinnamate (RI: 1.56, Sigma-Aldrich, 112372-100G) until imaging. Imaging was done using the Aurora airy beam LSFM system equipped with two multi-immersion objectives (Special Optics 0.4 NA @ 1.45 RI (RI 1.33-1.56; Mag 15.3x-17.9x) WD 12 mm FOV 870nm 1 µm axial resolution) and Hamamatsu Orca-Flash 4.0 V3 digital CMOS camera (M Squared Lasers Ltd.). Maximum intensity projection or volumetric data of these images was obtained using either Arivis Vision4D or Imaris Viewer V9. Segmentation of each organoid was done using virtual reality (VR) software SyGlass.

mRNA library preparation and sequencing and bioinformatics
Library preparation was done using the Illumina Stranded mRNA Prep Ligation (20040534, Illumina) with 14 cycles under final PCR amplification and samples were indexed with IDT for Illumina RNA UD Indexes Set B, Ligation (20040554, Illumina) for multiplexing. Clean up steps were automated using the King Fisher FLEX (18-5400620, Thermo Scientific). Library concentration was checked using the QuantIT 1X dsDNA HS Assay Kit (Q33232) and library size and quality were checked using the 5200 Fragment Analyzer™ 12-capillary (M3510AA, Agilent) using the DNF-930 dsDNA Reagent Kit, 75 bp – 20,000 bp (500 Samples) (Part # DNF-930-K0500, Agilent). A library input of 1.25 nM was sequenced using NovaSeq 6000 Sequencing System (20012850, Illumina). Alignment was done using STAR align and read counts were extracted using featureCounts (Dobin et al., 2012) (Liao et al., 2013). All reference genomes were from the Ensembl database (Mouse GRCm38 with the GTF annotation (release 101)). Analysis of RNAseq data was done on R using DEseq2, ggplot2, EnhancedVolcano, and pheatmap. Z-score is calculated based on substracting the mean TPM values of all samples in each gene from the TPM value for each sample for each gene divided by the standard deviation of the TPM values per gene across all samples.

$$\text{z-score: } \frac{Gene\ TPM_{(sample)} - Gene\ TPM_{(mean\ of\ all\ samples)}}{standard\ deviation}$$

Supplemental references

Alsafadi, H.N. (2022). IWT: Interactive Well Transformer. Zenodo. 10.5281/zenodo.7071741.

Dobin, A., Davis, C.A., Schlesinger, F., Drenkow, J., Zaleski, C., Jha, S., Batut, P., Chaisson, M., and Gingeras, T.R. (2012). STAR: ultrafast universal RNA-seq aligner. Bioinformatics *29*, 15-21. 10.1093/bioinformatics/bts635.

Eenjes, E., Mertens, T.C.J., Buscop-van Kempen, M.J., van Wijck, Y., Taube, C., Rottier, R.J., and Hiemstra, P.S. (2018). A novel method for expansion and differentiation of mouse tracheal epithelial cells in culture. Scientific Reports *8*, 7349. 10.1038/s41598-018-25799-6.

Liao, Y., Smyth, G.K., and Shi, W. (2013). featureCounts: an efficient general purpose program for assigning sequence reads to genomic features. Bioinformatics *30*, 923-930. 10.1093/bioinformatics/btt656.

Renier, N., Wu, Z., Simon, David J., Yang, J., Ariel, P., and Tessier-Lavigne, M. (2014). iDISCO: A Simple, Rapid Method to Immunolabel Large Tissue Samples for Volume Imaging. Cell *159*, 896-910. https://doi.org/10.1016/j.cell.2014.10.010.

Rock, J.R., Onaitis, M.W., Rawlins, E.L., Lu, Y., Clark, C.P., Xue, Y., Randell, S.H., and Hogan, B.L.M. (2009). Basal cells as stem cells of the mouse trachea and human airway epithelium. *106*, 12771-12775. 10.1073/pnas.0906850106 %J Proceedings of the National Academy of Sciences.

You, Y., Richer, E.J., Huang, T., and Brody, S.L. (2002). Growth and differentiation of mouse tracheal epithelial cells: selection of a proliferative population. American journal of physiology. Lung cellular and molecular physiology *283*, L1315-1321. 10.1152/ajplung.00169.2002.
